# Supplementary material for: Distribution equality as an optimal epidemic mitigation strategy
Source: Sci Rep. 2022 Jun 21;12:10430. doi: 10.1038/s41598-022-12261-x (PMC9210068; doi:10.1038/s41598-022-12261-x)
Supplement: Supplementary file 1 — Supplementary Information. [file 41598_2022_12261_MOESM1_ESM.pdf]

Distribution equality as an optimal epidemic mitigation strategy

Supplementary Material

Adar Hacohen, Reuven Cohen, Sol Efroni, Ido Bachelet & Baruch Barzel

March 31, 2022

Contents

1 Modeling the SIR epidemic spread and its mitigation **1**

1.1 SIR model 1

1.2 SIR model with treatment 3

1.3 Normalized equations 4

1.4 The human flux matrix 6

2 Dissemination of therapeutics **8**

2.1 Overview 8

2.2 Optimization 8

2.3 Normalized flow equations 10

2.4 Setting the local demands 12

3 Implementation **15**

3.1 Mobility networks and epidemic parameters 15

3.2 Numerical analysis 15

4 Heterogeneity in network-based distribution **17**

5 Additional tests **18**

5.1 Vaccine-based mitigation 21

6 Simulating COVID-19 **22**

1 Modeling the SIR epidemic spread and its mitigation

1.1 SIR model

To model epidemic spreading via air travel[1] we used a local susceptible-infected-recovered (SIR) model with diffusive coupling, following the formulation presented in Ref.[2]. In this framework each node n ($n = 1, \ldots, N$) represents a local population of M_n individuals, of which $S_n(t)$ are in the susceptible state $\mathcal{S}$, $I_n(t)$ are infected ($\mathcal{I}$) and $R_n(t)$ are recovered ($\mathcal{R}$), hence $S_n(t) + I_n(t) + R_n(t) = M_n$ for all t. Within each node, we assume a well-mixed population that locally follows SIR dynamics,[3–6] namely

$$
\begin{aligned}
\mathcal{I} + \mathcal{S} &\xrightarrow{\alpha} 2\mathcal{I} \\
\mathcal{I} &\xrightarrow{\beta} \mathcal{R},
\end{aligned}
\tag{1.1}
$$

where α and β are the infection and recovery rates, respectively. The coupling between two populations n and m is mediated by the flux of incoming/outgoing travelers via air-traffic F_{nm}, quantifying the number of individuals flying from m to n per day, namely $F_{nm} = F_{n \leftarrow m}$. This form of epidemic spreading gives rise to the following dynamic equations[2,7]

$$
\frac{\mathrm{d}S_n}{\mathrm{d}t} = -\alpha \frac{S_n(t) I_n(t)}{M_n} + \sum_{m=1}^{N} \Big(w_{nm} S_m(t) - w_{mn} S_n(t) \Big) \tag{1.2}
$$

$$
\frac{\mathrm{d}I_n}{\mathrm{d}t} = \alpha \frac{S_n(t) I_n(t)}{M_n} - \beta I_n(t) + \sum_{m=1}^{N} \Big(w_{nm} I_m(t) - w_{mn} I_n(t) \Big) \tag{1.3}
$$

$$
\frac{\mathrm{d}R_n}{\mathrm{d}t} = \beta \mathcal{I}_n(t) + \sum_{m=1}^{N} \Big(w_{nm} R_m(t) - w_{mn} R_n(t) \Big). \tag{1.4}
$$

The first term/s on the r.h.s. capture the processes of infection - proportional to the product of susceptible and infected individuals - and recovery, proportional to $I_n(t)$. The summation terms describe the diffusion of $\mathcal{S}, \mathcal{I}$ or $\mathcal{R}$ individuals between local populations, where

$$
w_{nm} = \frac{F_{nm}}{M_m} \tag{1.5}
$$

is the per-capita flux from m to n, hence, *e.g.*, $w_{nm} S_m(t)$ is the volume of susceptible passengers leaving m and entering n per day.

Finally, we introduce an invasion threshold ε, which *activates* the local SIR dynamics only if the infected population rises above an ε fraction of the local population. We apply this by adding a sigmoidal function[2]

$$\sigma(x) = \frac{(x/\varepsilon)^h}{1 + (x/\varepsilon)^h} \tag{1.6}$$

to the relevant equation terms, providing

$$\frac{\mathrm{d}S_n}{\mathrm{d}t} = -\alpha \frac{S_n(t) I_n(t)}{M_n} \sigma\left(\frac{I_n(t)}{M_n}\right) + \sum_{m=1}^{N} \Big(w_{nm} S_m(t) - w_{mn} S_n(t) \Big) \tag{1.7}$$

$$\frac{\mathrm{d}I_n}{\mathrm{d}t} = \alpha \frac{S_n(t) I_n(t)}{M_n} \sigma\left(\frac{I_n(t)}{M_n}\right) - \beta I_n(t) + \sum_{m=1}^{N} \Big(w_{nm} I_m(t) - w_{mn} I_n(t) \Big) \tag{1.8}$$

$$\frac{\mathrm{d}R_n}{\mathrm{d}t} = \beta I_n(t) + \sum_{m=1}^{N} \Big(w_{nm} R_m(t) - w_{mn} R_n(t) \Big). \tag{1.9}$$

Hence the process of infection is locally initiated (*i.e. turned on*) only when $I_n(t)/M_n$ exceeds ε, capturing an *invasion* of n.

Invasion threshold. The rational behind this threshold is to correct for an artifact that is otherwise unavoidable in our differential equation-based modeling. To understand this, let us first consider a stochastic realization of an SIR outbreak. It is seeded with a single infected individual, which then transmits the pathogen to its network neighbors. Quite often, due to the random nature of the spreading process, this initial seed may recover before it has the opportunity to transmit. This is the case even if the disease is in the pandemic phase with $\mathcal{R}_0 > 1$, as long as the number of infection individuals - the *seed* - is small. Indeed, the sharp distinction between $\mathcal{R}_0$ above or below unity is based on the mean-field analysis, relevant when the system is seeded with a macroscopic number of infections. Under such conditions the *law of large numbers* ensures a predictable decay ($\mathcal{R}_0 < 1$) or proliferation ($\mathcal{R}_0 \geq 1$). With a small number of infections, however, the disease may taper of, simply by chance, before it has the opportunity to *invade* the network.

This realistic behavior, however, is deeply ingrained in the stochastic nature of real pandemics, especially at their early stages, but overlooked in the deterministic setting of our SIR equations. Indeed, under the differential equation-based modeling, even an infinitesimal seed $\delta I_n \to 0$ is guaranteed to instigate a local pandemic spread with 100% certainty. Once, however, the seed grows sufficiently, such that I_n is no longer of order one or less, the mean-field solution kicks-in, and a stochastic elimination of the intial outbreak is no longer probable. This is precisely the role of $\sigma(x)$, which only activates out deterministic spread when

$$\frac{I_n(t)}{M_n} > \varepsilon. \tag{1.10}$$

This rationale provides clear guidelines for selecting ε. Assuming a total of N destinations, that together account for the global population Ω, we have the average population being $\langle M \rangle = \Omega/N$. Seeking $I_n(t) \gtrsim 1$, and substituting M_n in (1.10) by the average $\langle M \rangle$, we arrive at

$$\varepsilon \gtrsim \frac{N}{\Omega}, \tag{1.11}$$

ensuring an invasion of at least few individuals before the outbreak successfully penetrates a node.

1.2 SIR model with treatment

We now consider drug distribution, which divides the infected population into two separate groups, treated, who received the drug $\mathcal{Q}$, and untreated, who have not yet acquired it, hence

$$\mathcal{I} = \mathcal{I}^{\mathrm{T}} \cap \mathcal{I}^{\mathrm{U}}, \tag{1.12}$$

namely the infected population is constructed from the unity of treated and untreated infected individuals. We assume that uninfected individuals do not seek or receive treatment, *i.e.* no preventive measures, and hence the susceptible state $\mathcal{S}$ remains undivided. Similarly, once recovered the treated and untreated individuals reach the same state - which is non susceptible and non infectious - hence we also do not distinguish between treated and untreated individuals within the $\mathcal{R}$ population. Consequently, the possible transitions in each node are now driven by

$$\begin{aligned}
\mathcal{I}^{\mathrm{U}} + \mathcal{Q} &\overset{\rho}{\to} \mathcal{I}^{\mathrm{T}} \\
\mathcal{I}^{\mathrm{U}} + \mathcal{S} &\overset{\alpha}{\to} 2\mathcal{I}^{\mathrm{U}} \\
\mathcal{I}^{\mathrm{T}} + \mathcal{S} &\overset{\alpha}{\to} \mathcal{I}^{\mathrm{T}} + \mathcal{I}^{\mathrm{U}} \\
\mathcal{I}^{\mathrm{U}} &\overset{\beta}{\to} \mathcal{R} \\
\mathcal{I}^{\mathrm{T}} &\overset{\zeta}{\to} \mathcal{R},
\end{aligned} \tag{1.13}$$

where ρ is the drug consumption rate, β is the recovery rate from the disease untreated, as appears in (1.1), and $\zeta > \beta$ is the recovery rate under treatment.

We arrive at the following four equations

$$\frac{\mathrm{d}S_n}{\mathrm{d}t} = -\alpha \frac{S_n I_n}{M_n} \sigma \left(\frac{I_n}{M_n} \right) + \sum_{m=1}^{N} \Big(w_{nm} S_m - w_{mn} S_n \Big) \tag{1.14}$$

$$\frac{\mathrm{d}I_n^{\mathrm{U}}}{\mathrm{d}t} = \alpha \frac{S_n I_n}{M_n} \sigma \left(\frac{I_n}{M_n} \right) - \beta I_n^{\mathrm{U}} - \rho I_n^{\mathrm{U}} + \sum_{m=1}^{N} \Big(w_{nm} I_m^{\mathrm{U}} - w_{mn} I_n^{\mathrm{U}} \Big) \tag{1.15}$$

$$\frac{\mathrm{d}I_n^{\mathrm{T}}}{\mathrm{d}t} = -\zeta I_n^{\mathrm{T}} + \rho I_n^{\mathrm{U}} + \sum_{m=1}^{N} \Big(w_{nm} I_m^{\mathrm{T}} - w_{mn} I_n^{\mathrm{T}} \Big) \tag{1.16}$$

$$\frac{\mathrm{d}R_n}{\mathrm{d}t} = \zeta I_n^{\mathrm{T}} + \beta I_n^{\mathrm{U}} + \sum_{m=1}^{N} \Big(w_{nm} R_m - w_{mn} R_n \Big) \tag{1.17}$$

where

$$I_n = I_n^{\mathrm{U}}(t) + I_n^{\mathrm{T}}(t) \tag{1.18}$$

is the total infected population - treated and untreated - in n.

The consumption rate ρ depends on the drug availability in n, being zero in the limit of extreme shortage and approaching unity if there is sufficient availability to meet the demand. Hence we denote the total number of available drug doses in n by $Q_n(t)$, and its per-capita availability by

$$\varphi_n(t) = \frac{Q_n(t)}{I_n^{\mathrm{U}}(t)}, \tag{1.19}$$

in which the denominator represents the fraction of n's population that at time t continues to seek treatment. We then define the rate ρ as

$$\rho(\varphi_n) = \begin{cases} \varphi_n & \text{for} \quad \varphi_n < 1 \\ 1 & \text{for} \quad \varphi_n \geq 1 \end{cases} = \min\{\varphi_n, 1\}, \tag{1.20}$$

which in Eqs. (1.15) and (1.16) ensures that drug doses are consumed as long as there is local demand, *i.e.* $Q_n(t) < I_n^{\mathrm{U}}(t)$, while avoiding excess consumption when $Q_n(t) \geq I_n^{\mathrm{U}}(t)$, a case in which n has more available doses than required for its currently untreated population.

To close the set of equations (1.14) - (1.17) we must track the local availability of drugs $Q_n(t)$ at all locations. This is determined by our dissemination strategy, either Max-flow or Egalitarian, as detailed in Supplementary Section 2. Once selected, the dissemination strategy provides us with the daily influx $L_{n\leftarrow}(t)$ of drugs into n at each day t (doses per unit time). Starting from $t \geq t_{\mathrm{R}}$, our response time, we write

$$\frac{\mathrm{d}Q_n}{\mathrm{d}t} = \theta(t - t_{\mathrm{R}})L_{n\leftarrow}(t) - \rho(\varphi_n)I_n^{\mathrm{U}}(t), \tag{1.21}$$

where the first term represents the incoming drugs, as dictated by our dissemination scheme, and the second term captures their consumption. The Heavyside step-function

$$\theta(x) = \begin{cases} 0 & x < 0 \\ 1 & x \geq 0 \end{cases} \tag{1.22}$$

ensures dissemination only begins at $t = t_{\mathrm{R}}$.

1.3 Normalized equations

Next we rewrite Eqs. (1.14) - (1.17) for the normalized populations $s_n(t) = S_n(t)/M_n$, $j_n^{\mathrm{U}}(t) = I_n^{\mathrm{U}}(t)/M_n$, $j_n^{\mathrm{T}}(t) = I_n^{\mathrm{T}}(t)/M_n$ and $r_n(t) = R_n(t)/M_n$, obtaining

$$\frac{\mathrm{d}s_n}{\mathrm{d}t} = -\alpha s_n j_n \sigma(j_n) + \sum_{m=1}^{N} \left(\frac{M_m}{M_n} w_{nm} s_m - w_{mn} s_n \right) \tag{1.23}$$

$$\frac{\mathrm{d}j_n^{\mathrm{U}}}{\mathrm{d}t} = \alpha s_n j_n \sigma(j_n) - \beta j_n^{\mathrm{U}} - \gamma \rho j_n^{\mathrm{U}} + \sum_{m=1}^{N} \left(\frac{M_m}{M_n} w_{nm} j_m^{\mathrm{U}} - w_{mn} j_n^{\mathrm{U}} \right) \tag{1.24}$$

$$\frac{\mathrm{d}j_n^{\mathrm{T}}}{\mathrm{d}t} = -\zeta j_n^{\mathrm{T}} + \gamma \rho j_n^{\mathrm{U}} + \sum_{m=1}^{N} \left(\frac{M_m}{M_n} w_{nm} j_m^{\mathrm{T}} - w_{mn} j_n^{\mathrm{T}} \right) \tag{1.25}$$

$$\frac{\mathrm{d}r_n}{\mathrm{d}t} = \beta j_n^{\mathrm{U}} + \zeta j_n^{\mathrm{T}} + \sum_{m=1}^{N} \left(\frac{M_m}{M_n} w_{nm} r_m - w_{mn} r_n \right), \tag{1.26}$$

where $j_n = j_n^{\mathrm{U}}(t) + j_n^{\mathrm{T}}(t)$.

Assuming a negligible fraction of one-directional trips, *i.e.* that immigration accounts for a marginal part of the overall international mobility, we can write $F_{nm} = F_{mn}$, namely that, on average, the number of passengers flying daily from m to n is the same as those flying from n to m (see Supplementary Section 3). This enables us, using (1.5) to write

$$F_{nm} = F_{mn} \Rightarrow w_{nm} M_m = w_{mn} M_n, \tag{1.27}$$

which allows us to further simplify Eqs. (1.23) - (1.26), bringing them to their final normalized form

$$\frac{\mathrm{d}s_n}{\mathrm{d}t} = -\alpha s_n j_n \sigma(j_n) + \sum_{m=1}^{N} A_{nm} \left(s_m - s_n \right) \tag{1.28}$$

$$\frac{\mathrm{d}j_n^{\mathrm{U}}}{\mathrm{d}t} = \alpha s_n j_n \sigma(j_n) - \beta j_n^{\mathrm{U}} - \rho j_n^{\mathrm{U}} + \sum_{m=1}^{N} A_{nm} \left(j_m^{\mathrm{U}} - j_n^{\mathrm{U}} \right) \tag{1.29}$$

$$\frac{\mathrm{d}j_n^{\mathrm{T}}}{\mathrm{d}t} = -\zeta j_n^{\mathrm{T}} + \rho j_n^{\mathrm{U}} + \sum_{m=1}^{N} A_{nm} \left(j_m^{\mathrm{T}} - j_n^{\mathrm{T}} \right) \tag{1.30}$$

$$\frac{\mathrm{d}r_n}{\mathrm{d}t} = \beta j_n^{\mathrm{U}} + \zeta j_n^{\mathrm{T}} + \sum_{m=1}^{N} A_{nm} \left(r_m - r_n \right), \tag{1.31}$$

where

$$A_{nm} = \frac{M_m}{M_n} w_{nm} = w_{mn} \tag{1.32}$$

is the normalized human flux matrix.

Finally, the normalized Eq. (1.21), takes the form

$$\frac{\mathrm{d}q_n}{\mathrm{d}t} = \theta(t - t_{\mathrm{R}}) l_{n\leftarrow}(t) - \rho(\varphi_n) j_n^{\mathrm{U}}(t), \tag{1.33}$$

where $q_n(t) = Q_n(t)/M_n$ is the normalized drug availability, set to $q_n(t) = 1$ when there are sufficient doses for the entire population M_n, and

$$l_{n\leftarrow}(t) = \frac{L_{n\leftarrow}(t)}{M_n} \tag{1.34}$$

is the normalized influx, measuring incoming doses as a fraction of n's potential demand. Note that φ_n in (1.19) and hence ρ in (1.20) remain unchanged following this normalization, *i.e.* $\varphi_n = Q_n(t)/I_n^{\mathrm{U}}(t) = q_n(t)/j_n^{\mathrm{U}}(t)$.

1.4 The human flux matrix

To construct A_{nm} from data we denote the mobility rate by[2]

$$\mu = \frac{\sum_{m,n=1}^{N} F_{nm}}{\sum_{n=1}^{N} M_n}, \tag{1.35}$$

in which the numerator quantifies the number of individuals flying per day and the denominator equals to the total population, therefore providing the daily fraction of individuals who seek air travel. This allows us to estimate a local population M_n by[2]

$$M_n = \frac{1}{\mu} \sum_{k=1}^{N} F_{kn}, \tag{1.36}$$

assuming that the total number of people departing from n, *i.e.* $\sum_{k=1}^{N} F_{kn}$, represents a μ fraction of n's total population, M_n. We can now write

$$w_{nm} = \frac{F_{nm}}{M_m} = \mu \frac{F_{nm}}{\sum_{k=1}^{N} F_{km}} \tag{1.37}$$

and

$$\frac{M_m}{M_n} = \frac{\sum_{k=1}^{N} F_{km}}{\sum_{k=1}^{N} F_{kn}}, \tag{1.38}$$

providing

$$A_{nm} = \mu \frac{F_{nm}}{\sum_{k=1}^{N} F_{kn}}, \tag{1.39}$$

which allows us to extract A_{nm} directly from the mobility data (F_{nm}, μ).

Summary - SIR dynamics

Normalized equations for epidemic spreading with treatment:

$$\frac{\mathrm{d}s_n}{\mathrm{d}t} = -\alpha s_n j_n \sigma(j_n) + \sum_{m=1}^{N} A_{nm}\left(s_m - s_n\right) \tag{1.40}$$

$$\frac{\mathrm{d}j_n^{\mathrm{U}}}{\mathrm{d}t} = \alpha s_n j_n \sigma(j_n) - \beta j_n^{\mathrm{U}} - \rho(\varphi_n) j_n^{\mathrm{U}} + \sum_{m=1}^{N} A_{nm}\left(j_m^{\mathrm{U}} - j_n^{\mathrm{U}}\right) \tag{1.41}$$

$$\frac{\mathrm{d}j_n^{\mathrm{T}}}{\mathrm{d}t} = -\zeta j_n^{\mathrm{T}} + \rho(\varphi_n) j_n^{\mathrm{U}} + \sum_{m=1}^{N} A_{nm}\left(j_m^{\mathrm{T}} - j_n^{\mathrm{T}}\right) \tag{1.42}$$

$$\frac{\mathrm{d}r_n}{\mathrm{d}t} = \beta j_n^{\mathrm{U}} + \zeta j_n^{\mathrm{T}} + \sum_{m=1}^{N} A_{nm}\left(r_m - r_n\right) \tag{1.43}$$

$$\frac{\mathrm{d}q_n}{\mathrm{d}t} = \theta(t - t_{\mathrm{R}}) l_{n\leftarrow}(t) - \rho(\varphi_n) j_n^{\mathrm{U}}(t), \tag{1.44}$$

extracting A_{nm} from (1.39), $\rho(\varphi_n)$ from (1.20) and $l_{n\leftarrow}(t)$ from the relevant commodity flow algorithm (Max-flow/Egalitarian; Supplementary Section 2).

The relevant parameters: Passenger fluxes are characterized by F_{nm} and μ, evaluated from the flight data • Disease/therapeutic parameters α, β and ζ are selected to represent different epidemiological scenarios, from benign to severe.

2 Dissemination of therapeutics

2.1 Overview

To examine our dissemination strategies we used the commodity flow framework[8] to obtain the optimal shipping strategy. We consider the air-traffic network as our underlying *flow network*, upon which the single *source* node s must ship the therapeutic in sufficient quantities, to meet all initial demands $D_n(0)$ of all *target* nodes $n = 1, \dots, N$. Each link is characterized by a non-negative carrying capacity B_{nm}, capturing the maximal volume of the therapeutic that can be transferred from m to n through that link per unit time (day). Our goal is to meet the demand of all nodes in an optimal fashion, *i.e.* optimizing for Max-flow or Egalitarian, within the shipping constraints imposed by B_{nm}.

2.2 Optimization

At $t = 0$ we select a source node s, and set the initial demands of all nodes to $D_n(0)$, $n = 1, \dots, N$, capturing the number of individual therapeutic (drug or vaccine) doses required at n. Different strategies for setting $D_n(0)$ are discussed bellow. Denoting the volume of doses that are actually shipped from m to n on day t by $L_{nm}(t)$, we seek to maximize the total number of doses shipped each day from s. Under the egalitarian strategy, we also wish for these doses to be disseminated as evenly as possible across all nodes. Together, this translates to

$$\textbf{Maximize} \ \Big(L_{s\to}(t) + H \sum_{n=1}^{N} \lambda(t) D_n(t) \Big), \tag{2.1}$$

where

$$L_{n\to}(t) = \sum_{m=1}^{N} \big(L_{mn}(t) - L_{nm}(t) \big) \tag{2.2}$$

is the net outgoing flux of drug doses *from* n at time t, and

$$L_{n\leftarrow}(t) = -L_{n\to}(t) = \sum_{m=1}^{N} \big(L_{nm}(t) - L_{mn}(t) \big) \tag{2.3}$$

is the incoming flux *to* n, as appears in Eq. (1.21).

The second term in (2.1) captures the level of homogeneity in each dissemination step t. First, we denote by $\lambda_n(t) = L_{n\leftarrow}(t)/D_n(t)$ the fraction of n's demand that was supplied to n at time t. We then use this to obtain

$$\lambda(t) = \min_{\substack{n=1 \\ n \neq s}}^{N} \Big\{ \lambda_n(t) \Big\} \tag{2.4}$$

which is upper-bounded by the *least* supplied node at time-step t. Under a perfectly ho-

mogeneous spread of the therapeutic we have a uniform $\lambda_n(t)$, *i.e.* $\lambda_n(t) = \lambda(t)$ for all n, and hence $L_{s\to}(t) = \sum_{n=1}^{N} \lambda(t) D_n(t)$. Under these conditions the second term of (2.1) has a comparable contribution to the optimization as that of the first. In contrast, an uneven dissemination will lead to some $\lambda_n(t)$ being smaller, bounding the value of $\lambda(t)$, and consequently decreasing the target function (2.1). For example, a sequential supply pattern, in which nodes are supplied serially, is characterized by one or more destinations with $\lambda_n(t) = 0$ at each shipping instance. Indeed, such dissemination scheme first rapidly fills up the demand of few nodes, then turns to fill up that of the rest. Such supply strategy, while potentially fast, is highly uneven and has $\lambda(t) = 0$ at most time-steps. Equation (2.1) penalizes such inequality by having the second term vanish.

Therefore, the first term in (2.1) optimizes for Max-flow, while the second term enhances Egalitarian dissemination. The relative role of these two competing target functions is governed by the *homogeneity coefficient* H, whose value determines whether we prioritize flow volume ($H \to 0$) or flow equality ($H \approx 1$).

Following each day, the demands are updated as

$$D_n(t+1) = D_n(t) - L_{n\leftarrow}(t), \tag{2.5}$$

subtracting the incoming supply from the previous day's demand, thus obtaining the remaining demand at $t+1$. The maximization of (2.1) is subject to three constraints that must be satisfied for all t:

$$0 \le L_{nm}(t) \le B_{nm} \qquad \text{For all } n, m = 1, \dots, N \tag{2.6}$$

$$0 \le L_{n\leftarrow}(t) \le D_n(t) \qquad \text{For all } n = 1, \dots, N, n \neq s \tag{2.7}$$

$$L_{n\leftarrow}(t) \ge \lambda(t) D_n(t) \qquad \text{For all } n = 1, \dots, N, n \neq s \tag{2.8}$$

The first constraint (2.6) ensures that all daily shipments from m to n do not exceed the carrying capacity B_{nm}; the second constraint (2.7), with $D_n(t)$ taken from (2.5), restricts nodes from accumulating therapeutics in excess of their remaining demand, therefore upper-bounding the total incoming doses at day t, by the current demand $D_n(t)$. Constraint (2.8) is only relevant in case $H \neq 0$ in (2.1). Its goal, in accordance with Eq. (2.4), is to ensure all nodes receive at the least a portion $\lambda(t)$ of their demand at every time-step, avoiding a supply pattern in which few nodes receive their demand early on, while the rest are only supplied at a later stage. Under $H = 0$, the value of $\lambda(t)$ is irrelevant in the optimization, and therefore the optimal solution may result in $\lambda(t) = 0$, allowing a selective, and hence unequal, supply pattern.

Carrying capacity. To construct the carrying capacities B_{nm} we used the empirical passenger fluxes F_{nm}, as obtained from the air travel data[9]. We assume that the daily volume of cargo shipped across the $m \to n$ route is proportional to the number of passengers F_{nm} for that route[10]. This allows us to write

$$B_{nm} = \kappa F_{nm}, \tag{2.9}$$

where κ is the cargo conversion coefficient, capturing the number of doses that can be shipped as cargo per each traveling passenger. Our results are not sensitive to the specific value of κ, as any re-scaling of B_{nm} (capturing doses/day) is equivalent to an arbitrary re-scaling of the time units, having no bearing on our essential findings. Moreover, it is also possible that shipping takes place on different routes altogether, and hence the shipping network may be distinct from the travel network. Fortunately, our results do not depend on the specific network structure, as we show, using an arbitrary random network in Supplementary Section 5.

2.3 Normalized flow equations

To couple the commodity flow equations with the normalized SIR dynamics of Eqs. (1.40) - (1.44) we seek the per-capita incoming fluxes $l_{n\leftarrow}(t) = L_{n\leftarrow}(t)/M_n$ (1.34) that appear on the r.h.s. of Eq. (1.44). Using (2.2) and (2.3) we write

$$l_{n\rightarrow}(t) = \sum_{m=1}^{N} \left(\frac{L_{mn}(t)}{M_n} - \frac{L_{nm}(t)}{M_n} \right) \tag{2.10}$$

and

$$l_{n\leftarrow}(t) = \sum_{m=1}^{N} \left(\frac{L_{nm}(t)}{M_n} - \frac{L_{mn}(t)}{M_n} \right), \tag{2.11}$$

which we further express as

$$l_{n\rightarrow}(t) = \sum_{m=1}^{N} \left(\frac{M_m}{M_n} l_{mn}(t) - l_{nm}(t) \right) \tag{2.12}$$

$$l_{n\leftarrow}(t) = \sum_{m=1}^{N} \left(l_{nm}(t) - \frac{M_m}{M_n} l_{mn}(t) \right), \tag{2.13}$$

where

$$l_{nm}(t) = \frac{L_{nm}(t)}{M_n} \tag{2.14}$$

are the per-capita (in n) incoming rates from m to n. In (2.12) and (2.13) the population ratio M_m/M_n can be evaluated from the data based on Eq. (1.36), providing

$$K_{mn} = \frac{M_m}{M_n} = \frac{\sum_{k=1}^{N} F_{km}}{\sum_{k=1}^{N} F_{kn}}, \tag{2.15}$$

expressed in terms of the empirically available human flux matrix F_{nm}. These normalized rates, $l_{n\leftarrow}(t)$ and $l_{n\rightarrow}(t)$, are subject to the shipping constraints, which can be rewritten by substituting (2.14) and (1.34) into (2.6) - (2.8), providing

$$0 \le l_{nm}(t) \le b_{nm} \qquad \text{For all } n, m = 1, \dots, N \tag{2.16}$$

$$0 \le l_{n\leftarrow}(t) \le d_n(t) \qquad \text{For all } n = 1, \dots, N, n \ne s \tag{2.17}$$

$$l_{n\leftarrow}(t) \ge \lambda(t) d_n(t) \qquad \text{For all } n = 1, \dots, N, n \ne s. \tag{2.18}$$

In these normalized expressions the carrying capacities are

$$b_{nm} = \frac{B_{nm}}{M_n} \tag{2.19}$$

and the per-capita demands are

$$d_n(t) = \frac{D_n(t)}{M_n}. \tag{2.20}$$

Finally, dividing both sides of Eq. (2.5) by M_n, we obtain

$$d_n(t+1) = d_n(t) - l_{n\leftarrow}(t), \tag{2.21}$$

and the optimization of Eq. (2.1) becomes

$$\textbf{Maximize } \Big(l_{s\rightarrow}(t) + H \sum_{n=1}^{N} \lambda(t) d_n(t) \Big). \tag{2.22}$$

Max-flow. To optimize for max-flow we set $H = 0$ in (2.22). Under these conditions the optimization scheme seeks to only maximize the net outflux from s, $l_{s\rightarrow}(t)$, namely obtain a shipping sequence in which the volume of therapeutics introduced at each time-step into the network is as high as possible. Such sequence may be, and in fact, typically is, highly uneven. For example, supplying the entire demand of few nodes, then proceeding to supply that of the remaining nodes. Such supply pattern, while globally efficient, may be very inefficient for the supply-delayed nodes.

Egalitarian. Setting $H \approx 1$ we now favor supply sequences that also satisfy constraint (2.18). Such optimization penalizes unequal supply patterns, since the value of $\lambda(t)$ is determined, at each iteration, by the minimally supplied node in the network, as expressed in (2.4). Hence the optimal dissemination seeks not just a high volume of drugs shipped daily, but also an even spread of that volume, for which $\lambda(t)$ is maximal.

Normalized carrying capacities. To construct b_{nm} from data we take B_{nm} from (2.9) and M_n from (1.36), which in (2.19) provides us with

$$b_{nm} = \kappa\mu \frac{F_{nm}}{\sum_{k=1}^{N} F_{kn}}. \tag{2.23}$$

Hence, b_{nm} depends on the passenger data, as captured by the fluxes F_{nm} and by the

mobility rate μ - parameters that have been already introduced in the construction of the SIR dynamics. It requires, in addition, the conversion ratio κ, which controls the absolute numbers of doses that can be carried by each shipping route.

Normalized capacity C_s. The overall shipping capacity of the air transportation network is governed by the conversion ratio κ, capturing the volume of cargo (in doses) that can be shipped across each route per human passenger. In our presentation, however, we used the normalized $C_s \propto \kappa$, to describe the network's capacity in more *natural* units. Consider the maximal number of doses that can potentially be shipped from the source in each day. Following the constraint (2.6) we can express this as

$$\max\big(L_{s\to}(t)\big) = \sum_{m=1}^{N} B_{ms}, \tag{2.24}$$

capturing the fact that even if our optimization achieves ideal shipping rates, the outgoing flux of drugs from s is capped by the carrying capacity of all of s's outbound flight routes. Dividing by the global population, $\Omega = \sum_{n=1}^{N} M_n$, we obtain the daily per-capita shipping potential of s, namely $C_s = \max(L_{s\to}(t))/\Omega$. We rewrite this as

$$C_s = \frac{\sum_{m=1}^{N} B_{ms}}{\sum_{n=1}^{N} M_n} = \kappa\mu \frac{\sum_{m=1}^{N} F_{ms}}{\sum_{n=1}^{N}\sum_{m=1}^{N} F_{nm}}, \tag{2.25}$$

where we used (2.9) and (1.36) to express the numerator and the denominator, respectively.

The normalized capacity (2.25) is, indeed, proportional to κ, with a multiplicative factor that is fully determined by the available mobility data (F_{nm}, μ). It is gauged to follow $C_s = 1$ in case the network can, under ideal conditions, achieve an outflux of order Ω, *i.e.* disseminate sufficient doses to supply the global demand per day. Note, that this interpretation of C_s is independent of s, hence a capacity of, *e.g.*, $C_s = 1$ has the same meaning regardless of the identity of the source node. For example, consider two source nodes s and s' whose absolute shipping capabilities are $\sum_{m=1}^{N} B_{ms} = 2\sum_{m=1}^{N} B_{ms'}$, namely s has twice the shipping capabilities of s'. Under any specific choice of κ we have $C_s = 2C_{s'}$, capturing the fact that, indeed, under similar conditions s will achieve twice the dissemination efficiency as compared to s'. Conversely, if we wish to compare performance under a preset C_s, as we do in the main text, we must set $\kappa' = 2\kappa$, compensating for the difference between the two distinct source nodes. Hence C_s allows us to compare different mitigation scenarios without being sensitive to the specific characteristics of each realization, for instance the arbitrary selection of s.

2.4 Setting the local demands

We considered three different strategies for assigning the initial demands $d_n(0)$:

Population based. We set the demand in each location based on that location's population, *i.e.* $D_n(0) = M_n$. Under the normalized flow dynamics this maps to $d_n(0) = 1$ for all nodes. In our formulation, since M_n in (1.36) is proportional each node's weighted degree, this is equivalent to a degree-based supply pattern.

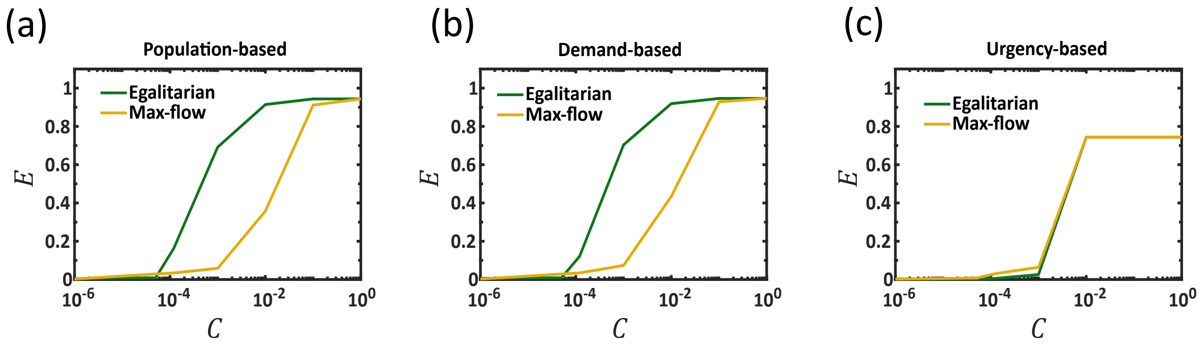

Figure S1: **Dissemination under different demand setting strategies**. The efficiency E vs. the normalized capacity C for max-Flow (yellow) and egalitarian (green), under the (a) Impact based; (b) Demand based; (c) Urgency based strategies. In all cases egalitarian shows an advantage over max-flow. For the urgency-based, the margin is much lower. This is because the urgency-based supply naturally mimics the time-evolution of the demands, in effect, transforming max-flow into an egalitarian form of dissemination.

Demand based. In the demand based strategy we used the projected infection rate at each destination to evaluate its anticipated demand. Hence, we first simulated the unmitigated epidemic via Eqs. (1.40) - (1.43) with $q_n(t) = 0$. Then, extracting $r_n(t \to \infty)$, we obtain the fraction of each population that will, at some point, become infected and require treatment. To meet this demand level we set $d_n(0) = r_n(t \to \infty)$, hence we supply each node based on its predicted demand.

Urgency based. Here, we implemented a dynamic strategy, in which the demands are updated in real-time, based on the states of infection ($j_n(t)$) and drug availability ($q_n(t)$) at each node. Therefore, we replace Eq. (2.5) with a dynamic update rule, following

$$d_n(t+1) = j_n(t) - q_n(t) - l_{n\leftarrow}(t). \tag{2.26}$$

Here the demand is updated at each time-step (day) based on the supply gaps observed in the previous time-step. This is captured by the difference between $j_n(t)$, capturing the demand level at time t, and the actual supply, which equals to the previous day supply $l_{n\leftarrow}(t)$ added to the already available drugs $q_n(t)$. The value of $j_n(t)$ and $q_n(t)$ are extracted from the SIR equations at the beginning of each day. In Eq. (2.26) time is measured starting from t_{R}, hence the initial demands are determined by the state of the epidemic at t_{R}.

Results obtained from Max-flow and Egalitarian with all three strategies are presented in Fig. S1. In all cases egalitarian (green) outperforms max-flow (yellow), as expected. Under the urgency-based scheme (Fig. S1c) the margin between max-flow and egalitarian becomes small. This is because this version of max-flow, by design, coordinates supply with demand. Indeed, each day's supply is set according to the demand left from the previous day at the relevant destination. Therefore, we arrive at a supply pattern that naturally mimics the spread of the pathogens, in a sense, rendering max-flow to deliver therapeutics in a similar fashion to that of egalitarian. In other words, the urgency-based scheme naturally leads to egalitarian supply, as it emulates the homogeneous spreading patterns of the pathogens. Yet, while the urgency-based strategy requires real-time update and tracking, egalitarian constructs the required homogeneous supply patterns quite simply from the start.

Summary - Flow dynamics

Normalized equations and constraints for flow optimization:

$$\textbf{Maximize}\ \Big(l_{s\rightarrow}(t) + H\lambda(t)\Big). \tag{2.27}$$

subject to

$$0 \le l_{nm}(t) \le b_{nm} \qquad \text{For all } n, m = 1, \dots, N \tag{2.28}$$

$$0 \le l_{n\leftarrow}(t) \le d_n(t) \qquad \text{For all } n = 1, \dots, N, n \neq s \tag{2.29}$$

$$l_{n\leftarrow}(t) \ge \lambda(t) d_n(t) \qquad \text{For all } n = 1, \dots, N, n \neq s. \tag{2.30}$$

where the preset demands $0 \le d_n(0) \le 1$ are updated via

$$d_n(t+1) = d_n(t) - l_{n\leftarrow}(t) \quad \bullet \quad d_n(t+1) = j_n(t) - q_n(t) - l_{n\leftarrow}(t) \ , \tag{2.31}$$

depending on the selected strategy in Sec. 2.4. The capacities b_{nm} are taken from (2.23), and the fluxes $l_{n\leftarrow}(t)$ and $l_{n\rightarrow}(t)$ are given by

$$l_{n\leftarrow}(t) = \sum_{m=1}^{N} \big(l_{nm}(t) - K_{mn} l_{mn}(t)\big) \quad \bullet \quad l_{n\rightarrow}(t) = \sum_{m=1}^{N} \big(K_{mn} l_{mn}(t) - l_{nm}(t)\big) \ , \tag{2.32}$$

with K_{mn} taken from (2.15).

To obtain the optimal solution for (2.27) we use an iterative approach (Fig. 1, main text): at each time step t we use linear optimization to maximize $l_{s\rightarrow}(t)$ under the three constraints. This provides us with day t's optimized shipping strategy, detailing the daily fluxes $l_{nm}(t)$ through all routes $m \rightarrow n$. With these fluxes we calculate the net influx into each node $l_{n\leftarrow}(t)$, and update the demands $d_n(t+1)$. We then use these updated demands to obtain the shipping strategy for the next day $t+1$, repeating the process until all demands vanish. The supply time $T_{\text{Supp},n}$ of node n is reached when $d_n(T_{\text{Supp},n}) = 0$ for the first time.

3 Implementation

3.1 Mobility networks and epidemic parameters

Passenger mobility network. We used the Global Mobility Network dataset[9] to extract the empirical fluxes F_{nm}. The dataset lists the daily travel of $\sim 8.91 \times 10^6$ passengers per day over the course of 365 days, *i.e.* $i = 1, \ldots, 365$, between $N = 1,292$ airports, linked through $38,377$ directional air-routes. The mean flux from m to n is thus captured by

$$F_{n \leftarrow m} = \frac{1}{365} \sum_{i=1}^{365} F_{n \leftarrow m}^{\{i\}} \tag{3.1}$$

where $F_{n \leftarrow m}^{\{i\}}$ is the number of passengers flying from m to n in day i. In our analysis we assume an almost symmetric flux matrix, as expressed in Eq. (1.27). Indeed we find that practically all links are bi-directional, and that in the vast majority of existing links we have $F_{n \leftarrow m} \approx F_{n \rightarrow m}$, a similar passenger flux in both directions. Therefore, to construct F_{nm} we used the symmetrized[2]

$$F_{nm} = \frac{F_{n \leftarrow m} + F_{n \rightarrow m}}{2}, \tag{3.2}$$

a network comprising $19,614$ bi-directional links, that represents a symmetric approximation of the empirical fluxes. Evaluating the local populations M_n as in (1.36) we obtained A_{nm} via (1.39). Following Ref.[2], we used $\mu = 1.7 \times 10^{-3}$ day^{-1} for the mobility rate.

Disease parameters. The disease parameters in (1.40) - (1.44) were set to $\beta = 0.285$ day^{-1} and $\alpha = 1.5\beta, 2\beta$ and 10β, providing a reproduction rate of $\mathcal{R}_0 = \alpha/\beta = 1.5, 2, 10$, as appears in the main text, covering different levels of contagion[3]. The treated population recovery rate is set to $\zeta = 1$ day^{-1}. For the penetration threshold $\sigma(j_n)$ in (1.6) we used Eq. (1.11) with $N \sim 10^3$ and $\Omega \sim 10^9$ to set $\varepsilon = 10^{-6}$; the saturation coefficient was set to $h = 8$. The response time $t_{\rm R}$ was set to be at the onset of the pandemic spread, evaluated as the time t when the infection levels have reached 10% of the projected peak infection (Fig. S2). The above represents our default parameters, used, *e.g.*, to produce Fig. 2c,d of the main text; in other figures we consistently vary some of these parameters, such as $t_{\rm R}$ or $\mathcal{R}_0$ as reported in the main text. Excluding the varied parameter in each panel, *e.g.*, $t_{\rm R}$ in Fig. 2i, the remaining parameters continue to be fixed at the above default values.

3.2 Numerical analysis

We used a 4th order Runge-Katta stepper to iteratively solve the SIR dynamics of Eqs. (1.40) - (1.44). To simulate an outbreak at node o we begin with an initial condition where $s_n(0) = 1$ and $j_n^{\rm U}(0) = j_n^{\rm T}(0) = r_n(0) = 0$, for all $n \neq o$. At node o we set $s_o(0) = 1 - \epsilon$ and $j_n^{\rm U}(0) = \epsilon$, having $\epsilon = 0.1$, to model a small local outbreak. The initial drug availability in (1.44) is set to $q_n(0) = 0$, hence, if unmitigated, $q_n(t)$ remains zero for all t. The epidemic reaches saturation for large t, as the derivatives on the l.h.s. of the equations vanish. We evaluate this by tracking $\dot{s}_n/s_n(t)$, which approaches zero towards the final stages of the

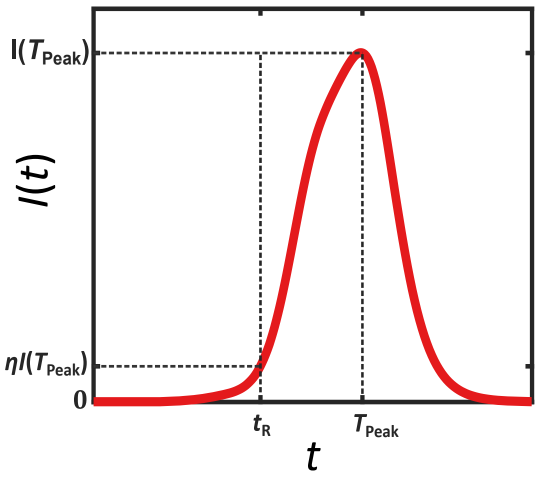

Figure S2: **Selecting the response time**. To select t_{R} we tracked the global level of infection, $I(t) = \sum_{n=1}^{N} I_n(t)$, vs. t, and set t_{R} to be the time where $I(t)$ has reached an η fraction of its peak value, namely $I(t_{\mathrm{R}}) = \eta I(T_{\mathrm{Peak}})$. Here we set $\eta = 0.1$.

spread. To numerically realize this limit we implemented the termination condition

$$\max_{n=1}^{N} \left| \frac{s_n(t_i) - s_n(t_{i-1})}{s_n(t_i)\Delta t_i} \right| < \xi, \tag{3.3}$$

where t_i is the time stamp of the ith Runge-Kutta step and $\Delta t_i = t_i - t_{i-1}$. This condition tracks the ratio between the *numerical derivative* $\dot{s}_n = s_n(t_i) - s_n(t_{i-1})/\Delta t_i$ and $s_n(t_i)$, guaranteeing that its maximum value over all nodes is smaller than the pre-defined termination parameter ξ. In our simulations we set $\xi = 10^{-3}$.

To model our mitigation we used linear optimization to maximize (2.27) in Eqs. (2.27) - (2.32). The optimization yields a supply sequence in the form of $l_{nm}(t)$, capturing the normalized daily therapeutic fluxes between all pairs of nodes $m \to n$. We then use (2.32) to extract the daily influx $l_{n\leftarrow}(t)$, quantifying the number of doses per capita that enter node n at each day t. The obtained $l_{n\leftarrow}(t)$ were then used as input in Eq. (1.44) to track the local drug availability in all nodes. While the Runge-Kutta stepper, implemented to solve the epidemic dynamics, incorporates variable time steps, typically much shorter than a single day, the optimization scheme provides $l_{n\leftarrow}(t)$ at a daily resolution. Therefore, to couple the commodity flow equations with the SIR dynamics we first applied the flow dynamics to extract $l_{n\leftarrow}(t)$. We then advanced the SIR equations day by day, updating $q_n(t)$ at the beginning of each day as

$$q_n(t+1) = q_n(t) + l_{n\leftarrow}(t), \tag{3.4}$$

after which $q_n(t)$ continues to evolve via (1.44). To be specific we used the following procedure:

- Set the response time t_{R} (days). As no drugs were supplied prior to this time point we set $l_{n\leftarrow}(t) = 0$ for $t = 0, \ldots, t_{\mathrm{R}}$.
- Use the commodity flow optimization to obtain $l_{n\leftarrow}(t)$ for $t = t_{\mathrm{R}} + 1, \ldots, T$, where T

is the overall supply time. The result is the inflow of drugs into n at each day t.

- Use variable time-step Runge-Katta to solve the SIR equations day by day. For example, at $t = 0$ we advance the equations until reaching the end of the first day at $t = 1$. At $t = 1$ we update $q_n(t)$ via (3.4), and set the resulting $q_n(1)$ as the new *initial condition* for the next day. We then continue to solve the SIR dynamics with initial condition $q_n(t)$ from $t = 1$ to $t = 2$. We continue the process until the epidemic reaches saturation, *i.e.* satisfies (3.3).

This process captures the continuous nature of the epidemic spread (variable time-step Runge-Kutta), and its coupling with the discrete daily bursts of the supply intervals. Note that since $l_{n\leftarrow}(t) = 0$ for $t < t_{\mathrm{R}}$ the day by day advancement of step 3 above only comes into effect when $t > t_{\mathrm{R}}$.

This coupling of the continuous-time SIR equations with the discrete-time commodity-flow dynamics becomes compatible in the limit where the discrete time steps are small compared to the SIR natural timescales. Under this limit the state of the pandemic does not change significantly between supply instances, and the discreteness is *smoothed* out. Here, as the pandemic timescales are of order of $\sim 10^2$ days, our day-by-day tracking of the supply, indeed, satisfies this condition.

4 Heterogeneity in network-based distribution

Consider the flow of a *unit* therapeutic from a single source node s, as it traverses all network pathways. With each step along its trajectory, it becomes diluted, first splitting among s's nearest neighbors, then its second neighbors and so on. In a random network, due to the locally tree-like structure, the number of nodes at distance $\mathbf{r}$ from the source grows exponentially as[11]

$$P(\mathbf{r}) \sim e^{\lambda \mathbf{r}} \tag{4.1}$$

where λ depends on the specific network topology. Hence, the therapeutic flux $l_{s\to}(t)$, exiting s daily, is dispersed among an exponentially growing number of nodes as it penetrates the network. For a typical node at distance $\mathbf{r}$ from s we, therefore, expect a supply rate of[12]

$$\mathbb{S}(\mathbf{r}) = l_{s\to}(t)e^{-\lambda \mathbf{r}} \tag{4.2}$$

an exponential decay that ensures a conservation of all doses flowing from s.

We can now use (4.2) to obtain the density function $P(\mathbb{S})$ to observe $\mathbb{S} \in (\mathbb{S}, \mathbb{S} + \mathrm{d}\mathbb{S})$ by transforming from the random variable $\mathbb{S}$ to the variable $\mathbf{r}$. We write

$$P(\mathbb{S})\,\mathrm{d}\mathbb{S} = P(\mathbf{r})\left|\frac{\mathrm{d}\mathbf{r}}{\mathrm{d}\mathbb{S}}\right|\mathrm{d}\mathbb{S}. \tag{4.3}$$

Taking $\mathbf{r}$ from (4.2) we have $\mathbf{r} = -\lambda^{-1}\ln\mathbb{S}$ and hence $|\,\mathrm{d}\mathbf{r}/\,\mathrm{d}\mathbb{S}| \sim \mathbb{S}^{-1}$. Using (4.1) and (4.2) we can also write $P(\mathbf{r}) \sim \mathbb{S}^{-1}$, hence, together we arrive at

$$P(\mathbb{S}) \sim \mathbb{S}^{-\nu}, \tag{4.4}$$

where $\nu = 2$, precisely the prediction of Fig. 3d of the main text.

This result is obtained under the configuration model ensemble, *i.e.* random network/weights. More generally, *e.g.*, in the presence of degree/weight correlations, the exponent ν can take different values. Similarly, if drugs are consumed as they flow through the network, a process neglected in the above derivation, the decay in (4.2) may become more rapid, again, impacting the exponent ν. Still, the power-law form of (4.4) will remain unaffected, albeit having $\nu \neq 2$. Indeed, as long as the network exhibits an exponential growth of the form (4.1), and the downstream dilution exhibits an exponential decay of the form (4.2), we expect a scale-free $P(\mathbb{S})$. To observe this, consider, for example, that the two exponential functions exhibit different growth/decay rates, *i.e.*

$$P(\mathbf{r}) \sim e^{\lambda_1 \mathbf{r}} \tag{4.5}$$
$$\mathbb{S}(\mathbf{r}) \sim e^{-\lambda_2 \mathbf{r}}. \tag{4.6}$$

Under these conditions we continue to have $|\,\mathrm{d}\mathbf{r}/\,\mathrm{d}\mathbb{S}| \sim \mathbb{S}^{-1}$, however $P(\mathbf{r})$ now follows $P(\mathbf{r}) \sim \mathbb{S}^{-\lambda_1/\lambda_2}$. Using these expressions in (4.3), we find that the scaling $P(\mathbb{S}) \sim \mathbb{S}^{-\nu}$ remains, only that now the scaling is

$$\nu = \frac{\lambda_1}{\lambda_2} + 1, \tag{4.7}$$

which may assume values different than 2. In case $\lambda_1 = \lambda_2$, Eq. (4.7) converges to our original derivation and provides $\nu = 2$. In Fig. 5d of the main text, we found that the empirical supply rates of the COVID-19 vaccine dissemination followed (4.4) with $\nu = 1.7$, consistent with (4.7) under $\lambda_1/\lambda_2 = 0.7$.

5 Additional tests

Our analysis of the two distributions $P(\mathbb{I})$ and $P(\mathbb{S})$ is rooted in the interplay between the network structure, *i.e.* the exponential growth of (4.1) and the spreading dynamics. For the pathogen, this exponential growth leads to the extremely short pathways, that help the

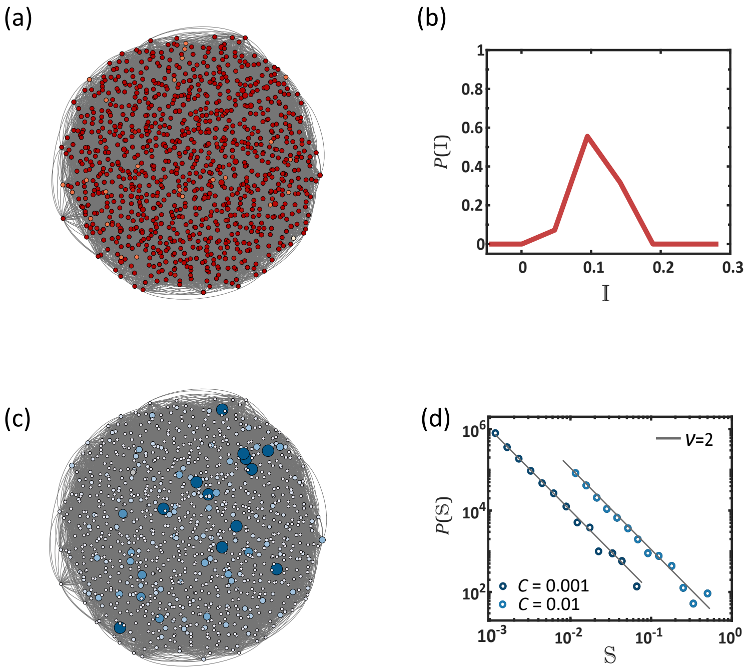

Figure S3: **Spreading patterns on a random network**. Our analysis is independent of the specific network upon which the pathogens/therapeutics spread. To show this we (a) tracked the pathogen spread on an Erdős-Rényi network. (b) As predicted, the spreading pattern is homogeneous, expressed by the bounded $P(\mathbb{I})$. (c) - (d) The theraputic spreads heterogeneously, following Eq. (4.4) with $\nu = 2$. Results are shown for two values of the shipping capacity C.

disease spread to the majority of nodes concurrently, resulting in the bounded $P(\mathbb{I})$. For the therapeutics it leads to the exponential dilution of (4.2), which, in turn, renders $P(\mathbb{S})$ scale-free. The crucial point is, that (4.1) is a quite generic characteristic of, practically all random networks, and therefore, as we explain below in detail, we expect our analysis to be largely independent of the specific network structure, or of the arbitrary selection of the source node.

To examine this more deeply, we consider the impact of the source node selection. In a heterogeneous network, *e.g.*, scale-free, different nodes may, in principle, have a distinct local environment, and hence one might expect that they would lead to a different dissemination outcome. Still, while such differences are local, capturing a node's immediate surrounding, they do not lead to different global dynamics. This is because the exponential growth rate λ in (4.1) captures a global network characteristic, which is largely independent of the source node. To understand this consider two highly distinct nodes: node 1 a hub, and node 2, a peripheral node. At $\mathbf{r} = 1$, these two nodes interact with a profoundly different environment, with 1 having many neighbors and 2 having just a few. However, as we advance further at $\mathbf{r} = 2, 3, \ldots$ the more distant neighbors of both 1 and 2 become statistically indistinguishable.[11] Therefore, the exponential growth rapidly converges to (4.1), with the network's global growth rate λ. Consequently, the global spreading patterns of the pathogens ($P(\mathbb{I})$) and the therapeutics ($P(\mathbb{S})$) are independent of (i) the specific network structure; (ii) the source of the disease outbreak; and (iii) the source of the therapeutic distribution. Below we examine each of these predictions (i) - (iii).

Network structure. To observe the fact that network structure has little impact on the supply/demand patterns we constructed an Erdős-Rényi network of $N = 1,000$ nodes with

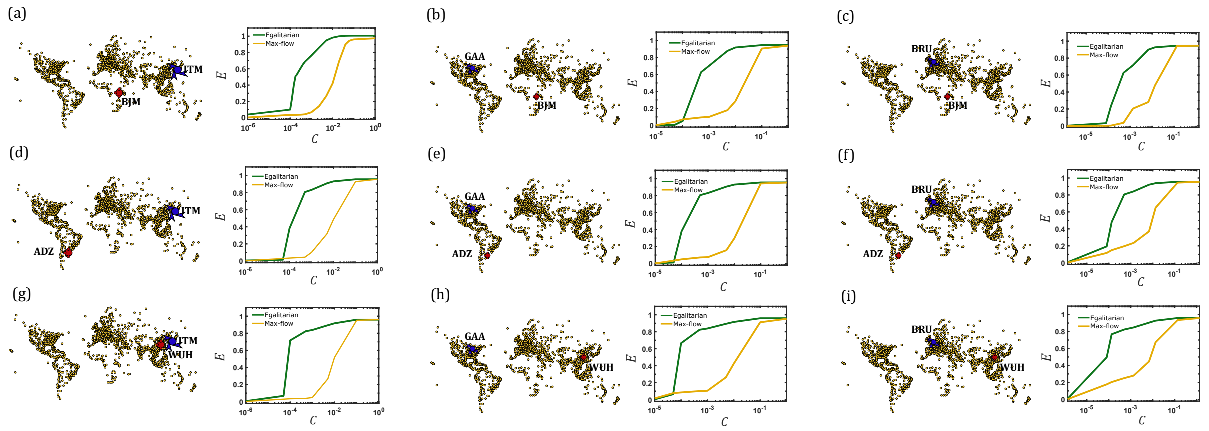

Figure S4: **Examining different outbreak and therapeutic source nodes**. We repeated our simulations for 9 different combinations of the outbreak origin o and the dissemination source s. We find that the specific choice of o, s has no impact on the observed mitigation efficiency.

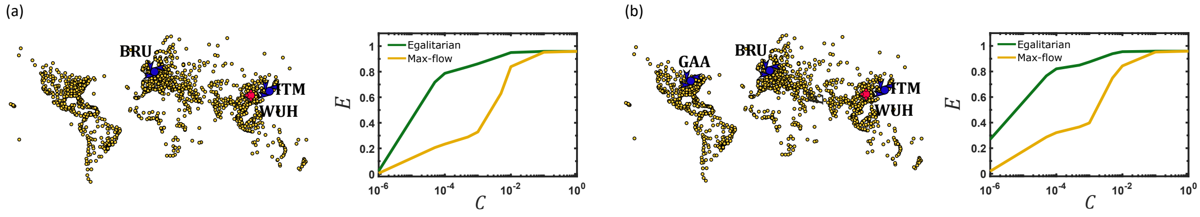

Figure S5: **Shipping from multiple sources**. (a) Splitting the dissemination between two sources improves the performance of both strategies, however egalitarian (green) continues to outperform max-flow (yellow). (b) Similar results observed under three sources.

average degree $\langle k \rangle = 15$, and link weights extracted from a normal distribution $\mathcal{N}(\mu, \sigma^2)$, setting $\mu = 1, \sigma^2 = 0.1$ (and discarding negative weights). This represents a fundamentally different network from the air-transportation network used in our main analysis, which has a scale-free topology and extremely heterogeneous weights. Still, as Fig. S3 clearly shows the pathogen/therapeutic distributions continue to follow our predicted patterns: the pathogens exhibit a homogeneous spread, and hence a concurrent global demand, while the therapeutics follow the predicted power-law ($\nu = 2$), depicting a highly unbalanced supply.

Outbreak/dissemination source. To complete our examination of the max-flow vs. egalitarian dissemination, we repeated our analysis this time selecting a set of 6 random assignments for the outbreak node o and the therapeutic source node s (Fig. S4). In all cases we examine the mitigation efficiency E vs. capacity C, *a là* Fig. 2h of the main text. As predicted, we observe no discernible difference between the resulting plots, as - indeed - our analysis is independent of the selection of source/outbreak nodes.

Multiple sources. In a real-world setting, the therapeutic may be distributed from more than one source s. Using multiple sources, can, quite naturally, enhance the performance of both strategies - max-flow and egalitarian. However, as long as the number of sources n is small, *i.e.* $n \ll N$, the egalitarian advantage is guaranteed. This is because, in a large network, each source continues to be surrounded by a tree-like expansion of the form (4.1), leading to the predicted supply/demand discrepancy. Of course, when the number of sources is increased, as we approach $n \lesssim N$, we have each node being in close proximity to one of the sources. Under these conditions, all nodes benefit from a similar supply rate - indeed

they all have a supplier in their neighborhood - and hence they are all equally treated. As a consequence, for all purposes, such conditions converge to our proposed egalitarian dissemination, and the max-flow/egalitarian distinction is rendered moot. In Fig. S5 we examine the impact of 2 or 3 distribution sources, finding that n has, indeed, little impact on the egalitarian advantage.

5.1 Vaccine-based mitigation

Egalitarian mitigation is designed around drug distribution, but continues to provide improved performance, albeit less dramatic, also in the case of vaccines. To examine this we implemented a susceptible-infected-vaccinated-recovered (SIVR) model, in which the disease is battled through vaccines rather than drugs. The resulting transitions are now

$$\mathcal{I} + \mathcal{S} \overset{\alpha}{\to} 2\mathcal{I} \tag{5.1}$$
$$\mathcal{S} + \mathcal{Q} \overset{\rho}{\to} \mathcal{V} \tag{5.2}$$
$$\mathcal{I} \overset{\beta}{\to} \mathcal{R}, \tag{5.3}$$

in which the second equation describes a susceptible individual receiving the vaccine $\mathcal{Q}$ and entering the vaccinated state $\mathcal{V}$. Vaccinated individuals, similar to recovered, can no longer infect or become infected. Analogically to the case of the drug dissemination, described in Eqs. (1.19) and (1.20), we evaluate the vaccination rate via

$$\varphi_n(t) = \frac{Q_n(t)}{S_n(t)}, \tag{5.4}$$

and

$$\rho(\varphi_n) = \begin{cases} \varphi_n & \text{for} \quad \varphi_n < 1 \\ 1 & \text{for} \quad \varphi_n \geq 1 \end{cases} = \min\{\varphi_n, 1\}, \tag{5.5}$$

in which $\varphi_n(t)$ is the vaccine availability as a fraction of the potential demand, here captured by the susceptible population.

Following a similar analysis to the one leading to the normalized SIR equations (1.40) - (1.44), we can now track the SVIR spread via

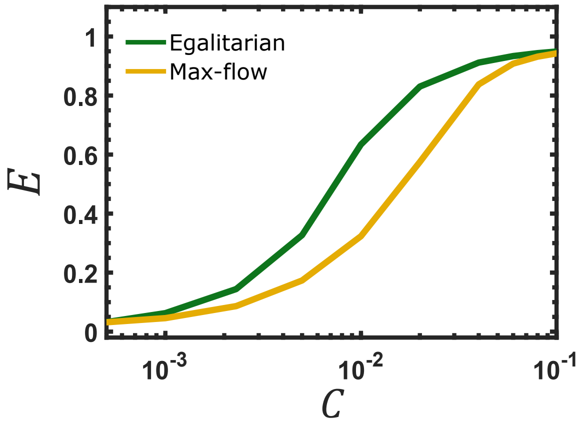

Figure S6: **Vaccine-based mitigation**. E vs. C as obtained from solving (5.6) - (5.10) under max-flow (yellow) and egalitarian (green) vaccine dissemination. Egalitarian performs better, albeit not as dramatically as in the case of drug dissemination.

$$\frac{\mathrm{d}s_n}{\mathrm{d}t} = -\alpha s_n j_n \sigma(j_n) - \rho(\varphi_n) s_n + \sum_{m=1}^{N} A_{nm} (s_m - s_n) \tag{5.6}$$

$$\frac{\mathrm{d}j_n}{\mathrm{d}t} = \alpha s_n j_n \sigma(j_n) - \beta j_n + \sum_{m=1}^{N} A_{nm} (j_m - j_n) \tag{5.7}$$

$$\frac{\mathrm{d}v_n}{\mathrm{d}t} = \rho(\varphi_n) s_n + \sum_{m=1}^{N} A_{nm} (v_m - v_n) \tag{5.8}$$

$$\frac{\mathrm{d}r_n}{\mathrm{d}t} = \beta j_n + \sum_{m=1}^{N} A_{nm} (r_m - r_n) \tag{5.9}$$

$$\frac{\mathrm{d}q_n}{\mathrm{d}t} = \theta(t - t_{\mathrm{R}}) l_{n\leftarrow}(t) - \rho(\varphi_n) s_n(t), \tag{5.10}$$

where s_n, j_n, v_n and r_n are the normalized $\mathcal{S}, \mathcal{I}, \mathcal{V}, \mathcal{R}$ populations, and q_n is the normalized vaccine availability. Simulating (5.6) - (5.10), in Fig. S6 we extracted the efficiency E vs. the shipping capacity C under both max-flow (yellow) and egalitarian (green). We find that egalitarian offers improved mitigation, however, the margin is smaller than that observed under drug distribution.

6 Simulating COVID-19

To track the COVID-19 scenario we added an additional compartments to our SIR modeling framework: the exposed individuals ($\mathcal{E}$), who have contracted the virus, but are still not infectious. The resulting transitions are now

$$
\begin{aligned}
\mathcal{I}^{\rm U} + \mathcal{Q} &\overset{\rho}{\rightarrow} \mathcal{I}^{\rm T} \\
\mathcal{I}^{\rm U} + \mathcal{S} &\overset{\alpha}{\rightarrow} \mathcal{I}^{\rm U} + \mathcal{E} \\
\mathcal{I}^{\rm T} + \mathcal{S} &\overset{\alpha}{\rightarrow} \mathcal{I}^{\rm T} + \mathcal{E} \\
\mathcal{E} &\overset{\gamma}{\rightarrow} \mathcal{I}^{\rm U} \\
\mathcal{I}^{\rm U} &\overset{\beta}{\rightarrow} \mathcal{R} \\
\mathcal{I}^{\rm T} &\overset{\zeta}{\rightarrow} \mathcal{R},
\end{aligned}
\tag{6.1}
$$

in which upon exposure a susceptible individual becomes exposed, later transitioning at a rate γ to the infectious state. Once again we use Eqs. (1.19) and (1.20) to evaluate the drug intake rate ρ. Finally, to account for the travel restrictions imposed during COVID-19, we do not allow the infected population to travel internationally, removing them from the diffusion terms of the dynamic equations. The resulting normalized SEIR equations are

$$\frac{\mathrm{d}s_n}{\mathrm{d}t} = -\alpha s_n j_n \sigma(j_n) + \sum_{m=1}^{N} A_{nm} (s_m - s_n) \tag{6.2}$$

$$\frac{\mathrm{d}e_n}{\mathrm{d}t} = \alpha s_n j_n \sigma(j_n) - \gamma e_n + \sum_{m=1}^{N} A_{nm} (e_m - e_n) \tag{6.3}$$

$$\frac{\mathrm{d}j_n^{\rm U}}{\mathrm{d}t} = \gamma e_n - \beta j_n^{\rm U} - \rho(\varphi_n) j_n^{\rm U} \tag{6.4}$$

$$\frac{\mathrm{d}j_n^{\rm T}}{\mathrm{d}t} = -\zeta j_n^{\rm T} + \rho(\varphi_n) j_n^{\rm U} \tag{6.5}$$

$$\frac{\mathrm{d}r_n}{\mathrm{d}t} = \beta j_n^{\rm U} + \zeta j_n^{\rm T} + \sum_{m=1}^{N} A_{nm} (r_m - r_n) \tag{6.6}$$

$$\frac{\mathrm{d}q_n}{\mathrm{d}t} = \theta(t - t_{\rm R}) l_{n\leftarrow}(t) - \rho(\varphi_n) j_n^{\rm U}(t), \tag{6.7}$$

where s_n, e_n, j_n and r_n are the normalized $\mathcal{S}, \mathcal{E}, \mathcal{I}, \mathcal{R}$ populations, and q_n is the normalized drug availability. The air-transportation fluxes A_{nm} are extracted from our mobility data, via Eq. (1.39), namely

$$A_{nm} = \mu \frac{F_{nm}}{\sum_{k=1}^{N} F_{kn}}, \tag{6.8}$$

allowing us to tune the volume of international travel by changing μ, in order to account for the observed reduction in air travel in response to the pandemic. In (6.4) and (6.5) we omitted the travel terms completely for infected individuals.

Evaluating the parameters. To construct a realistic simulation we collected data on the time-scales of the different transitions characterizing the COVID-19 disease cycle.[13] This provides us with $\gamma = 1/3$ days^{-1} and $\beta = 0.1$ days^{-1}, capturing the average 3 day

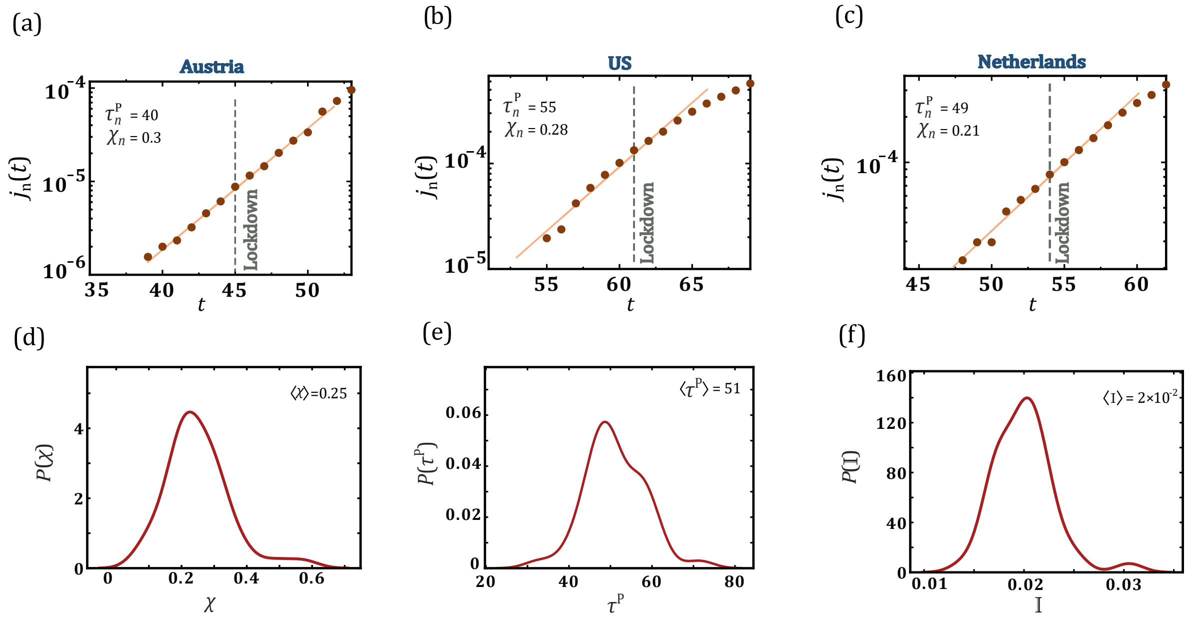

Figure S7: **Evaluating COVID-19 time-scales**. (a)-(c) The fraction of infected individuals $j_n(t)$ vs. t (circles) in three different destinations of the 41 tracked countries in Supplementary Dataset 1. Time $t = 0$ is set to January 22, 2020, the time of the initial outbreak in Wuhan, China. In each country we track the spread up to 3 days post lock-down (grey dashed lines), after which the exponential trend begins to break. Fitting the data to a shifted exponential of the form (6.9) we extract the growth rate χ_n and the delay τ_n^{P}. (d) The probability density $P(\chi)$ for a random country to have growth rate $\chi_n \in (\chi, \chi + \mathrm{d}\chi)$. (e) The probability density $P(\tau^{\mathrm{P}})$ for a random country to have delay $\tau_n^{\mathrm{P}} \in (\tau^{\mathrm{P}}, \tau^{\mathrm{P}} + \mathrm{d}\tau^{\mathrm{P}})$. (f) The probability density $P(\mathbb{I})$ for a random country to have infection rate $\mathbb{I}_n \in (\mathbb{I}, \mathbb{I} + \mathrm{d}\mathbb{I})$.

incubation period, and 10 day recovery time-line. To capture the reduction in air-travel we set $\mu = 0.85 \times 10^{-3}$ day^{-1}, a reduction of 50% as compared to normal circumstances.

The infection rate α cannot be directly measured, and we therefore evaluated it from the observed spreading dynamics. Hence, we collected data from the early stages of the pandemic in 41 different countries.[14] In each country we track the initial exponential rise in $j_n(t)$, prior to the instigation of lock-downs or quarantines (Fig. S7a-c, Supplementary Dataset 1). We then fit the observed data to a shifted exponential of the form

$$j_n(t) = j_n(0) e^{\chi_n (t - \tau_n^{\mathrm{P}})}, \tag{6.9}$$

in which χ_n is the observed growth rate of the infected population at n and τ_n^{P} is the pathogen delay, capturing the propagation time for the virus to reach n. The pre-factor $j_n(0)$ is extracted directly from the first data-point in each country's epidemic data. The distribution of χ_n across all 41 monitored countries is shown in Fig. S7d, a bounded distribution with mean growth rate of $\chi = 0.25$ days^{-1}, consistent with other similar analyses.[15] Next we implemented our SEIR disease cycle with the relevant parameters $\gamma = 1/3, \beta = 0.1$, under $q_n(t) = 0$, *i.e.* no vaccine, to seek the infection rate α than best matches the observed $\chi = 0.25$. This allows us to infer $\alpha = 0.62$ days^{-1}, completing our empirical extraction of all parameters required for Eqs. (6.2) - (6.7).

Extracting $P(\mathbb{I})$ **and** $P(\mathbb{S})$. The parameter τ_n^{P} in Eq. (6.9) captures the propagation time of the pathogen from the outbreak source o to the destination n. Hence, by fitting (6.9) to the observed spreading dynamics, we can extract the time needed for the pathogen to reach

each of our 41 tracked destinations. The *rate* by which the pathogens advance towards n is, therefore

$$\mathbb{I}_n = \frac{1}{\tau_n^{\mathrm{P}}}, \tag{6.10}$$

providing an empirical evaluation of Eq. (8) of the main text. In Fig. S7e we show $P(\mathbb{I})$ as obtained from our empirical pool of countries, finding that it is indeed bounded, indicating the homogeneous SARS-CoV-2 spreading patterns.

To obtain the vaccine supply patterns, we collected data[16] on the vaccination rate of 229 countries (Supplementary Dataset 2). We then measured the elapsed time τ_n^{S} from the initial vaccine dissemination (December 1,2020), until country n began its vaccination campaign. This helps us extract the supply rate of Eq. (9) in the main text via

$$\mathbb{S}_n = \frac{1}{\tau_n^{\mathrm{S}}}, \tag{6.11}$$

whose distribution is displayed in Fig. 5d of the main text.

References

[1] V. Colizza, A. Barrat, M. Barthélemy and A. Vespignani. The role of the airline transportation network in the prediction and predictability of global epidemics. *Proc. Natl. Acad. Sci. USA*, 103:2015, 2006.

[2] D. Brockmann and D. Helbing. The hidden geometry of complex, network-driven contagion phenomena. *Science*, 342:1337–1342, 2013.

[3] R. Pastor-Satorras, C. Castellano, P. Van Mieghem and A. Vespignani. Epidemic processes in complex networks. *Rev. Mod. Phys.*, 87:925–958, 2015.

[4] A. Barrat, M. Barthélemy and A. Vespignani. *Dynamical Processes on Complex Networks.* Cambridge University Press, Cambridge, 2008.

[5] P.S. Dodds and D.J. Watts. A generalized model of social and biological contagion. *Journal of Theoretical Biology*, 232:587–604, 2005.

[6] L. Hufnagel, D. Brockmann and T. Geisel. Forecast and control of epidemics in a globalized world. *Proc. Natl. Acad. Sci. USA*, 101:15124–9, 2004.

[7] L. Sattenspiel and K. Dietz. A structured epidemic model incorporating geographic-mobility among regions. *Mathematical Biosciences*, 128:71–91, 1995.

[8] R.K. Ahuja, T.L. Magnanti and J.B. Orlin. *Network flows: theory, algorithms and applications.* Prentice Hall, 1993.

[9] OAG Ltd. 2018-06-11. *https://www.oag.com/.*

[10] O. Woolley-Meza, C. Thiemann, D. Grady, J. J. Lee, H. Seebens, B. Blasius and D. Brockmann. Complexity in human transportation networks: A comparative analysis of worldwide air transportation and global cargo-ship movements. *The European Physical Journal B*, 84:589–600, 2011.

[11] M.E.J. Newman. *Networks - an introduction.* Oxford University Press, New York, 2010.

[12] B. Barzel and A.-L. Barabási. Universality in network dynamics. *Nature Physics*, 9:673 – 681, 2013.

[13] Yinon M Bar-On, Avi Flamholz, Rob Phillips, and Ron Milo. Sars-cov-2 (covid-19) by the numbers. *eLife*, 9, 2020.

[14] E. Dong, H. Du and L. Gardner. An interactive web-based dashboard to track COVID-19 in real time. *The Lancet Infectios Diseases*, 2020.

[15] D. Meidan, N. Schulmann, R. Cohen, S. Haber, E. Yaniv, R. Sarid and B. Barzel. *Nature Communications*, 12:220, 2021.

[16] https://ourworldindata.org
